# Ultrasonographic assessment of normal jugular veins in Standardbred horses

**DOI:** 10.1186/s12917-019-2104-5

**Published:** 2019-10-16

**Authors:** Maria Pia Pasolini, Giuseppe Spinella, Chiara Del Prete, Simona Valentini, Pierpaolo Coluccia, Luigi Auletta, Michele Greco, Leonardo Meomartino

**Affiliations:** 1 Department of Veterinary Medicine and Animal Production, University of Naples Federico II, Via a Federico Delpino, 1, 80137 Naples, Italy; 2 Department of Veterinary Medical Sciences, University Alma Mater of Bologna, via Tolara di Sopra 50, 40064 Ozzano dell’Emilia, BO Italy; 3 IRCCS SDN, via E. Gianturco 113, 80143 Naples, Italy; 4 Clevedale Veterinary Practice, Home Farm, Redcar, UK; 5 Interdepartmental Radiology Centre, University of Naples Federico II, Via Federico Delpino 1, 80137 Naples, Italy

**Keywords:** Equine, Italian Standardbred, Jugular veins, Phlebitis, Ultrasonography, Venous wall thickness

## Abstract

**Background:**

Ultrasonography (US) is the recommended imaging technique to evaluate jugular veins. This prospective randomized clinical study was designed to collect a series of B-mode US measurements of manually distended jugular veins in healthy Italian Standardbreds and to find possible correlations between ultrasound measurements and animal morphometric characteristics. Forty-two horses, eight males and 34 females (range 3–22 years; bodyweight 494.4 ± 41.7 kg), were included in the study. The diameters and wall thicknesses of both jugular veins were measured at three different sites of the neck. The differences in ultrasound measurements based on scans, age, gender, side, and site of the neck were evaluated by ANOVA or by the Kruskal-Wallis test. The effects of the morphometric measures on each ultrasound parameter were evaluated by MANOVA (*P* < 0.05).

**Results:**

The ultrasound measurements did not differ significantly between the three different sites or between genders; hence, they were pooled together in the results. On the transverse scan, the mean dorsoventral and lateromedial diameters were 1.58 ± 0.23 and 2.20 ± 0.25 cm, respectively; the mean superficial and deep wall thicknesses (SWT and DWT) were 0.07 ± 0.01 and 0.08 ± 0.01 cm, respectively. On the longitudinal scan, the mean dorsoventral diameter was 1.59 ± 0.26 cm, and the SWT and DWT were both 0.08 ± 0.01 cm. Neck length, from the caudal edge of the mandible to the thoracic inlet, was related to the dorsoventral diameter in both longitudinal and transverse scan and to the SWT and DWT in transverse scan, whereas height at the withers (measured with tape) and estimated weight were related to the wall thickness. Dividing the subjects into groups by age in years (“young” 3–7, “mature” 8–14, “old” > 14), differences were found for the lateromedial diameter in the transverse scan and the SWT on the longitudinal scan. The main limitation of this study was that only one operator performed the measurements.

**Conclusion:**

The US measurements of the jugular veins and their relationship with morphometric measures reported in this manuscript might be considered as guidelines both for early diagnosis and monitoring jugular vein abnormalities in healthy Italian Standardbred horses.

## Background

Jugular vein catheterizations and intravenous injections are often performed in horses both within hospital and field settings, and they may represent the most important predisposing factor for thrombophlebitis in the horse [1]. Nevertheless, thrombophlebitis has been observed in the absence of a known pre-existing vascular trauma [2].

The diagnosis of thrombophlebitis is usually based on clinical and ultrasonographic examination [1]. The use of ultrasounds (US) allows detecting thrombophlebitis, assessing vessel patency, characterizing the type of thrombus (cavitating or non-cavitating), selecting a site for treatment (i.e., aspiration) and monitoring the progression the disease [2]. Ultrasonographically, thrombophlebitis is characterized by wall thickening and presence of irregularly shaped hyperechoic luminal mass with homogenous echogenicity or, less frequently, inhomogeneous texture with scattered hyper- and/or hypo-echoic spots [2].

Ultrasonographic physiologic reference values for jugular veins in healthy horses, stratified by age groups, sex, and breeds, might be useful for both clinical procedures, diagnosis, and follow-up of vascular diseases. Specifically, in cases of bilateral jugular vein disease, it might not be possible to use the contralateral healthy vein for comparison. Unfortunately, to the authors’ knowledge, there are limited quantitative data collected in brightness- (B-) mode to provide guidelines in diagnosing subclinical vascular diseases, characterised by subtle prodromal wall ‘thickening’ in horses [3, 4]. Acute jugular vein thickening has been described as a two-fold hypoechogenic increase of the wall thickness, when compared to control veins [3]. Differences between breeds, as well as relationships between jugular vein diameter and morphometric measures, has been detected in cows [4] and might exist in horses. Also, ageing, as demonstrated in human beings and laboratory models, produces morphofunctional changes in veins, by inducing a reduced vasoconstrictive response, abnormal compliance and wall thickening, as well as reduction of the cross-sectional area [5–7].

This study aimed to collect a series of B-mode US measurements of the manually distended jugular veins in healthy Italian Standardbred horses and to evaluate their relationship with morphometric measures.

## Results

Measurements were obtained on eighty-four veins of 42 horses. The mean (± SD) weight, withers height, and neck length were 494.4 kg (±41.7), 159.4 cm (±5.0) and 51.9 (±5.5) cm, respectively. The mean ± SD, median, and range for each US measurement are reported in Table 1. The US measurements of diameters, thicknesses and vein perimeters were not different between the three measurement sites (CrS, MdS, and CaS) and between male and female horses; thus, such measurements were pooled together and the two aforementioned categories, i.e., neck site and sex, were not further considered. There were no differences between measurements by right and left veins, except for LDVVD, which resulted larger in the left vein (*P* = 0.0013).

**Table 1:** Mean ± standard deviation (SD), median, minimum and maximum values for each ultrasound measurement of the jugular veins, obtained in longitudinal and transverse scans. Sub-category values (mean ± SD) for side, weight, withers height and neck length, as identified by a standard least square model for effects’ leverage analysis, were reported

	Ultrasound parameters				Morphometric parameters					
		*Mean* ± SD *(Median; range) cm*	*Side*		*Weight* (494.44 ± 41.73 cm)		*Withers height* (159.40 ± 4.96 cm)		*Neck length* (51.89 ± 5.54 cm)	
*Longitudinal* *Scan*	Dorsoventral vein diameter (LDVVD)	1.59 ± 0.26 (1.61;0.96–2.28)	*left*	*right*	–		–		*< 52 cm*	*≥52 cm*
			1.65 ± 0.24	1.53 ± 0.27					1.51 ± 0.27	1.70 ± 0.21
	Superficial wall thickness (SWT)	0.08 ± 0.01 (0.08;0.05–0.11)	–		*≤490 kg*	*> 490 kg*	–		–	
					0.07 ± 0.01	0.08 ± 0.01				
	Deep wall thickness (DWT)	0.08 ± 0.01 (0.08;0.05–0.11)	–		–		–		–	
*Transverse scan*	Dorsoventral vein diameter (TDVVD)	1.58 ± 0.23 (1.57; 1.01–2.12)	–		–		–		*< 52 cm*	*≥52 cm*
									1.54 ± 0.23	1.61 ± 0.23
	Lateromedial vein diameter (LMVD)	2.20 ± 0.25 (2.21;1.43–2.80)	–		–		–		–	
	Superficial wall thickness (SWT)	0.07 ± 0.01* (0.07;0.04–0.10)	–		–		*≤159 cm*	*> 159 cm*	*< 52 cm*	*≥52 cm*
							0.073 ± 0.01	0.069 ± 0.01	0.07 ± 0.01	0.08 ± 0.01
	Deep wall thickness (DWT)	0.08 ± 0.01* (0.08;0.04–0.10)	–		–		*≤159 cm*	*> 159 cm*	*< 50 cm*	*≥50 cm*
							0.08 ± 0.01	0.07 ± 0.01	0.07 ± 0.01	0.08 ± 0.01
	Vein perimeter (VP)	5.65 ± 0.50 (5.67;4.52–6.62)	–		–		–		–	

* indicates significant differences between longitudinal and transverse scans (*P* < 0.05)

There was no significant difference between the LDVVD and the TDVVD, i.e. the scanning plane did not influence the measurement of the dorsoventral vein diameter. Both TDVVD and LDVVD were affected by neck length (*P* = 0.0151, *P* < 0.0001, respectively; split value 52 cm, for both).

The SWT and DWT were larger in the longitudinal scan than in the transverse scan (*P* < 0.0001; *P* = 0.0021, respectively). The SWT in longitudinal scan was affected by bodyweight (*P* = 0.0012; split value 490 kg). Both SWT and DWT resulted affected by neck length (*P* < 0.0001, split value 52 cm; *P* < 0.0001, split value 50 cm; respectively) and withers height (*P* = 0.0116, split value 159 cm; *P* = 0.0008, split value 159 cm; respectively) in the transverse plane. As shown in Table 1, the split value defines sub-categories. Weight, withers height and neck length did not show any significant effect on other measurements.

The subjects were divided into three age categories: young horses (3–7 years old), mature horses (8–14 years old) and old horses (> 14 years old). Eleven horses were included in the “young” group, 21 in the “mature”, and 10 in the “old”. The mean ± SD (median; range) of each US measurement for each age group are reported in Table 2. The SWT in longitudinal scan was significantly larger in the mature horses than both in the young (*P* = 0.0395) and the old (*P* = 0.0084) horses. The VP was significantly larger in both mature and old horses than in young horses (*P* < 0.0001). The LMDV was significantly larger in mature than in both young (*P* = 0.0001) and adult horses (*P* = 0.0099), there was also a significant difference between LMDV of adult and young horses (*P* = 0.0004). When considering the effect of morphometric measures within age groups, only SWT in longitudinal scan resulted influenced by bodyweight in old horses (*P* = 0.0091), with a reduction in its mean value in heavier horses.

**Table 2:** Ultrasonographic measurements of jugular veins, obtained in longitudinal and transverse scans, expressed as the mean ± SD (median; range) in the age sub-category groups. Different lowercase letters (a, b and c) indicate significant differences between groups (*P* < 0.05)

Scansion	Ultrasound Parameters	Age group		
		*Young (3–7 years old)*	*Mature (8–14 years old)*	*Old (> 14 years old)*
*Longitudinal* *Scan*	Dorsoventral vein diameter (LDVVD)	1.65 ± 0.16^a^ cm (1.72;1.11–1.85)	1.58 ± 0.27 ^a^ cm (1.61;0.96–2.28)	1.58 ± 0.28 ^a^ cm (1.59;0.98–2.27)
	Superficial wall thickness (SWT)	0.07 ± 0.01^a^ cm (0.08;0.06–0.09)	0.08 ± 0.01^b^ cm (0.08;0.05–0.1)	0.07 ± 0.01^a^ cm (0.07;0.05–0.11)
	Deep wall thickness (DWT)	0.08 ± 0.01^a^ cm (0.08;0.06–0.1)	0.08 ± 0.01^a^ cm (0.08-;0.06–0.11)	0.08 ± 0.01^a^ cm (0.08;0.05–0.11)
*Transverse scan*	Dorsoventral vein diameter (TDVVD)	1.54 ± 0.18 ^a^ cm (1.53; 1.2–1.9)	1.59 ± 0.23^a^ cm (1.58;1.01–2.12)	1.56 ± 0.25^a^ cm (1.55;1.08–2.12)
	Lateromedial vein diameter (LMVD)	2.02 ± 0.19^a^ cm (2.02;1.55–2.33)	2.10 ± 0.25^b^ cm (2.21;1.43–2.76)	2.29 ± 0.24^c^ cm (2.29;1.86–2.8)
	Superficial wall thickness (SWT)	0.07 ± 0.01^a^ cm (0.07;0.05–0.08)	0.07 ± 0.01 ^a^ cm (0.07;0.04–0.11)	0.07 ± 0.01^a^ cm (0.07;0.04–0.09)
	Deep wall thickness (DWT)	0.08 ± 0.01^a^ cm (0.08;0.05–0.1)	0.08 ± 0.01^a^ cm (0.08;0.05–0.1)	0.07 ± 0.01 ^a^ cm (0.07;0.04–0.1)
	Vein perimeter (VP)	5.65 ± 0.50^a^ cm (5.67;4.52–6.62)	6.02 ± 0.64^b^ cm (6.09;3.89–7.64)	6.17 ± 0.65^b^ cm (6.25;4.84–7.78)

## Discussion

To the best of our knowledge, the literature does not include any studies about US distended jugular vein diameters in healthy Italian Standardbred horses. Based on the results of this study, dorsoventral venous diameters of up to 2.3, lateromedial venous diameters of up to 2.8 cm, perimeters of up to 6.6 cm and both superficial and deep wall thicknesses of up to 0.11 cm can be expected in a population of average-sized healthy Italian Standardbred horses. In contrast, venous diameters of up to 2.4 cm can be expected in a population of average-sized Warmblood and Freiberger horses, with slightly larger veins found in Warmblood horses [4].

Reference or guideline values are an invaluable tool for clinicians and radiologists to promptly detect abnormalities in the studied organs, in human medicine as much as in veterinary medicine [8]. The US values reported in this study may be guidelines for maximal venous filling. Determination of the jugular vein diameters may confirm the clinical finding of congestion of the jugular vein or hypovolemic shock. In horses, normal jugular veins should appear collapsed, when no pressure is applied to interrupt blood flow and, hence, to determine the vein filling [9]. Jugular vein congestion, i.e., distension without any external pressure on it, may be related to venous flow obstruction, raised intrathoracic or central venous pressure [10]. Distension with “rope-like” appearance can also be detected during thrombophlebitis [11]. Differently, hypovolemia leads to delayed jugular vein filling [12], which might be confirmed by US recording smaller venous diameters after the 15 s compression. In humans, US measurements, such as the height-width ratio of the internal jugular vein (IJV) or the so-called “corrected” IJV longitudinal length, have been correlated with central venous pressure and blood loss [13, 14]. In human medicine, literature about US measurements of the external jugular vein is limited [15], whereas measurements of IJV are of importance, as ultrasonography is the preferred modality for the diagnosis of phlebectasia and is often used as a guide for interventions performed via the IJV [16, 17]. Indeed, in human medicine, ultrasonographic assessment of IJV diameters, area and distensibility, i.e., in response to the Valsalva or other manoeuvre, are gaining importance in the clinical setting for a number of reasons [18]. For example, it has been used to correctly identify dyspnoeic patients as affected by congestive heart failure [19–21]. A ratio between measurements at rest and after the Valsalva manoeuvre greater than 1.5 has been considered as diagnostic for phlebectasia in children [22]. Moreover, as said, it can be used to monitor hemodynamic parameters, such as the central venous pressure or to predict fluid therapy responsiveness [23–25]. Hence, the determination of reliable guideline values for jugular vein measures in Standardbred horses of various age groups might be useful to determine the prognosis after the resolution of thrombophlebitis, as well as for detection and follow-up of other venous or systemic diseases. Nonetheless, the best ultrasonographic measures for diagnosing vascular or vascular-associated diseases in horses has not yet been determined, and the guideline values reported in here should be looked as a starting point for future research

In the present study, specific inclusion criteria were the absence of intravenous injection in the last 3 months and the absence of any abnormalities of the jugular veins at clinical and US evaluation. The healing process of the vein wall after venepuncture was studied both macroscopically and with light-microscopy [26]. Lesions experimentally induced in ponies were not macroscopically visible 5 weeks after the injection, independent of the needle size [26]. Similarly, in horses, wall thickening after jugular vein catheterization resolved as soon as the catheter was removed, and no wall lesions were evident at the US examination 4 weeks after catheter insertion [27]. The US evaluation as an inclusion criterion was mandatory, since subclinical alterations may go undetected at the sole clinical evaluation, and its use for clinical, forensic or experimental applications has been highly recommended [28, 29].

Ultrasonographic examination of the jugular vein should be performed in both longitudinal and transverse planes [30, 31], eventually focusing on the region of interest. Bain [30] suggested initially evaluating the vein in a transverse plane and then in a longitudinal plane, which is useful to demonstrate linear anatomic relationships, such as the linear extent of a thrombus along the course of the jugular vein. Schwarzwald and Jenni [4] measured the vein diameters in the transverse plane parallel to the ultrasound beam at the widest diameter, bisecting the vein into two equal parts. In our experience, the dorsoventral diameters in longitudinal and transverse scans did not differ, but both superficial and deep thicknesses were larger in longitudinal scans than in transverse scans. Measurements in the transverse scan may result in poor quality images due to the artefacts produced by refraction from the sidewall [31]. The presence of hair may create US artefacts, as well; but cleaning the area from debris and soaking the hair with alcohol is routinely sufficient for good image quality [4, 9, 30].

In the cow, Braun et al. [32] reported an increase in the vein diameter from cranial to caudal. Differently, the jugular veins examined in the present study showed a constant diameter, as well as wall thickness, throughout their lengths.

In our sample, no significant differences were identified between male and female horses; however, only eight male horses were included in the study. A larger number of males in further studies should be included to confirm this result. On the other hand, in human medicine, the IJV diameter is independent of gender [33, 34].

In our sample, measurements obtained by the right and left veins did not differ, except for the dorsoventral diameter in the longitudinal scan, which resulted significantly larger in the left vein. In human medicine, cannulation of the left IJV is associated with a perceived increased level of difficulty when compared with the contralateral IJV. Such increased difficulty has been explained by either a smaller diameter or a more anterior position relative to the corresponding carotid artery of the left IJV [35]. However, contrasting data have been reported regarding the asymmetry of the IJV in human beings [40,4142]. Indeed, some studies have reported that right-side IJV dimensions were greater than left-side dimensions [35–37]. In contrast, in other studies, diameters did not differ at rest, whereas significant differences were observed in children during the Valsalva manoeuvre [34, 38]. Also, in cows, little differences were detectable between the right and the left external jugular veins [32]. Thus, further studies would be necessary to demonstrate if the major left dorsoventral diameter observed in the longitudinal scan, in the horses of the present study is due to a real anatomical difference or a different pressure of the probe, maintained by a right-handed operator. Moreover, it should be considered that clipping hair coat would have allowed obtaining the best quality of details, hence preventing any distortion of the image acquired by applying too much pressure on the transducer.

Karazincir et al. [38] and Verghese et al. [39] reported a positive correlation between age and IJV diameter in children ranging from birth to 6 years of age. Eksioglu et al. [34] provided IJV reference values for children/adolescents ranging from birth to 18 years of age and reported a strong correlation between age and vein measures only in the birth-2 year age group. The strength of correlation decreased with increasing age groups. In adult humans, Mortensen et al. [33] reported that there was no significant correlation between age and IJV diameter in 32 adult participants. On the contrary, Magnano et al. [6] found that IJV cross-sectional area increases with aging, and such occurrence is more evident in the left side and in males. In the present study, short transverse axis and vein perimeters were significantly larger in mature and old horses than in young horses, whereas superficial wall thickness in the longitudinal scan was significantly larger in adult horses than in both young and old horses. While the strength of the correlation between age and vein measurements may be related to the growth period in the human pediatric population [34], in old and mature horses, aging and senescence of the vein wall may be related to changes in vein elasticity and dimension. Nevertheless, larger and more homogenous samples of horses belonging to different age groups should be considered to confirm our results. Moreover, the superficial wall thickness in the longitudinal scan was influenced by both age and body weight, and we did not consider supplementary multivariate analysis since groups’ numerousness would have been further reduced.

The range of jugular vein diameters found by Schwarzwald and Jenni [4] in a population of average-sized Warmblood and Freiberger horses varied considerably; within each population, there was no significant relationship between jugular vein diameter and body size estimated by girth length. Therefore, in their opinion, prediction of the jugular vein diameter in every single horse based on breed and girth length is inaccurate. Our data added new information to the relationship between morphometric parameters and jugular vein size. Neck length was related to the dorsoventral vein diameter both in the longitudinal and transverse scan, and to SWT and DWT in the transverse scan. These latter two US measurements resulted to be influenced by withers height, as well. Calculated weight influenced significantly only the superficial wall thickness in the longitudinal scan. Approximately 50 cm, 159 cm and 490 kg were the split values for neck length, withers height and weight, respectively, for the sampled breed, for all the US measurements considered.

Ultrasonography is well accepted as an accurate and reliable method for measuring the size of peripheral blood vessels [33]. Ultrasonography of the jugular veins requires temporary occlusion to image the lumen of the vessel. Thus, to reduce the influence of this subjective parameter, the same researcher at the same site, the inlet of the thorax, always performed compression for an uninterrupted time of 15 s for each measurement. However, a limit of the present study is that measures may be influenced by the degree of the compression exerted [40]. A double-blind evaluation by two operators could assess the potential error produced by the slight compression of the vein beneath the skin during probe positioning. Another possible source bias during US measurements is the operator-dependence of this imaging exam; hence, we choose to have all acquisitions and all measurements performed by the same experienced veterinary radiologist. It would be interesting to explore, in further studies, intra- and inter-operators agreement on US jugular vein examination in horses, as well as in other species.

## Conclusions

In conclusion, these reported US values could be applied for the follow-up of patients with indwelling venous catheters, the early diagnosis and monitoring of abnormalities of the jugular veins in Italian Standardbred horses, in relation to age and size. Based on our results, transverse scan allows collecting more measurements in terms of diameters and calculated perimeter, but the longitudinal plane resulted more reliable for thickness measurement, for the absence of lateral acoustic shadow artefact provided by sidewalls.

## Methods

### Animals

Italian Standardbred horses, considered healthy based on physical examination and clinical history, were enrolled in the study. Inclusion criteria were: (1) no history of intravenous injection in the jugular veins in the last 3 months and (2) absence of any abnormalities at inspection, palpation and ultrasound examination of the jugular veins. Forty-two horses, eight males and 34 females (median age 11 years, range 3–22; mean ± standard deviation – SD – bodyweight 494.4 ± 41.7 kg), were included in the study.

The horses were privately owned and kept at three different livery yards. Subjects were included in this study after written informed consent was obtained from the owners. The study was performed on the premises where the animals were regularly housed.

Sex, age, withers height (cm), neck length (cm), and body weight (kg) were recorded. Neck length was measured as the distance from the caudal edge of the mandible to the caudal end of the jugular groove at the thoracic inlet. Bodyweight was calculated using the formula: weight (kg) = heart girth (cm)^2^ x body length (cm) / 11,877 [41]. The heart girth was obtained with a measuring tape placed around the trunk, passing immediately behind the elbow and the withers [42]. Body length was measured from the point of the shoulder to the *tuber ischii*.

### Techniques

All examinations were performed in a standing position; horses were not sedated and only gently restrained by a skilled equine handler.

All veins were imaged by the same experienced radiologist using a portable US device equipped with a multi-frequency linear transducer (LA435 VET, 6–18 MHz operating frequency) set at 10 MHz (ESAOTE MyLab™ 30GoldVet).

**Fig. 1:**
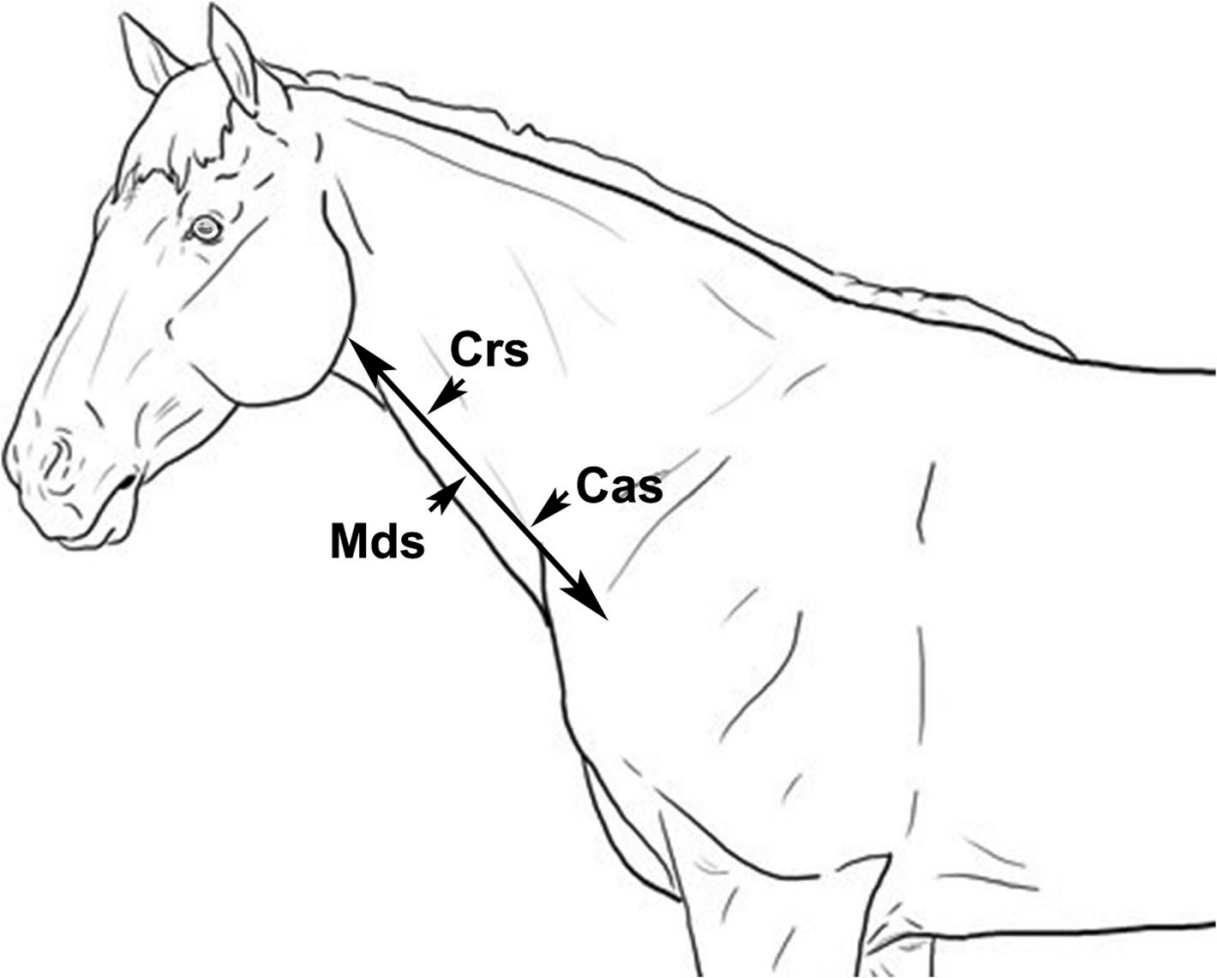
Schematic drawing of the sites on which the vein diameter (VD) and the wall thickness (WT) of the left and right jugular veins were measured: half-way between the mandibular angle and the thoracic inlet (Mid Site: MdS); half-way between the mandibular angle and the MdS (Cranial site: CrS); half-way between the MdS and the thoracic inlet (Caudal site: CaS).Design Professor Leonardo Meomartino (our co-author)

All procedures were repeated for the right and the left jugular vein and the side to be scanned first was randomly selected. The jugular grooves were cleaned with 90% ethanol. Hair was not clipped, as requested by owners, hence the hair coat of the region was first saturated with 90% ethanol and then a coupling gel was applied to ensure adequate contact with the transducer. A digital compression was applied at the thoracic inlet to dilate the jugular lumen and the entire vein was scanned from the thoracic inlet towards the head, to rule out any clinically undetectable alteration, which would have led to the exclusion of the vein from the study. Subsequentially, the vein diameter (VD) and the wall thickness (WT) were measured on both longitudinal and transverse planes at three different sites: half-way between the mandibular angle and the thoracic inlet (Mid Site: MdS), half-way between the mandibular angle and the MdS (Cranial site: CrS) and half-way between the MdS and the thoracic inlet (Caudal site: CaS) (Fig. 1).

The maximum cross-sectional diameter of the external jugular vein was measured after 15 s of manual occlusion [4]. Each scan at each of the three neck points was performed as a new acquisition, thus releasing the pressure on the jugular vein for at least 30 s; no sliding movements of the probe were performed between the different neck points, and alcohol saturation and coupling gel application were repeated for each scan. The best obtainable acquisitions in the transverse and the longitudinal plane at each neck point were fixed and recorded for offline measurement. Each of the measurement described in the following paragraphs was repeated three times on the same image by the same experienced veterinary radiologist, and the mean values were used in the analysis.

**Fig. 2:**
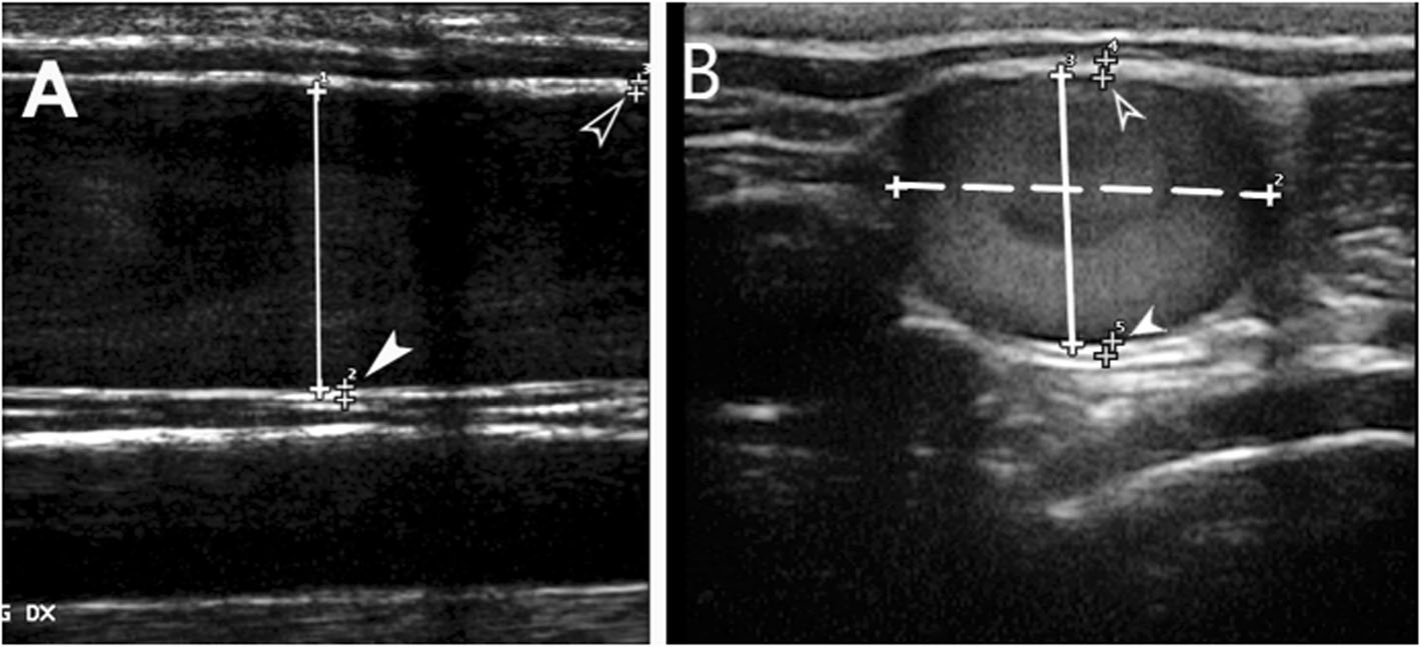
Representative ultrasonographic image of the jugular lumen diameter and wall thickness measurements in the longitudinal scan (**a**) and transverse scan (**b**): dorsoventral vein diameter (DVVD; solid line); lateromedial vein diameter (LMVD; dotted line); superficial (SWT; empty arrowhead) and deep wall thickness (DWT; white arrowhead)

The VD was measured as the distance from the leading edge of the intima-lumen interface of the near wall to the leading edge of the lumen-intima interface of the far wall at the widest diameter. On the longitudinal scan, the dorsal-ventral vein diameter (LDVVD) was measured (Fig. 2a). On the transverse scan, the dorsoventral vein diameter (TDVVD) and the lateromedial vein diameters (LMVD) were measured (Fig. 2b).

Superficial (SWT) and deep wall thicknesses (DWT) in both transverse and longitudinal scans were measured as the intima-media complex, i.e., the distance from the leading edge of the lumen-intima interface of the wall to the leading edge of the media-adventitia interface of the wall (Fig. 2a-b) [43].

The vein perimeters (VP) were calculated by the following mathematic formula:

$$ \mathrm{VP}=2\uppi \sqrt{\frac{{\left(\frac{\mathrm{d}}{2}\right)}^2+{\left(\frac{\mathrm{D}}{2}\right)}^2}{2}} $$

where d is the smallest (TDVVD) and D is the largest (LMVD) jugular vein diameter, in the transverse scan.

### Statistical analysis

All data were recorded on an electronic spreadsheet (Excel® 2011, Microsoft® for Mac) before importing them into commercial software for statistical analysis (JMP® 8.0.2, SAS Institute, Inc.). For each US measurement, the mean ± standard deviation (SD), median and range were calculated with the Reference Value Advisor set of visuals macro for Microsoft Excel®, as described by Geffré et al. [44].

Normality was tested by the Shapiro-Wilk’s *W* test. Parametric tests were used for normally distributed measures (vein diameters and perimeters), whereas non-parametric tests were used for not normally distributed measures (wall thickness). Mean vein diameters, vein perimeters, and wall thickness were compared between transverse and longitudinal scans, genders and side (right or left) by pooled Student’s *t* test or Mann-Whitney’s *U* test, according to distribution, and between age groups and measurement site by ANOVA or Kruskal-Wallis test, as appropriate; post hoc analysis was performed with Tukey’s *HSD* test.

Effects of the morphometric measures (weight, withers height, and neck length) on each ultrasound parameter were evaluated by multivariate analysis of variance (MANOVA). The “identity” response was chosen to test which of the model’s effects, i.e., the weight, withers height and neck length, would affect a significant deviation from the global mean of the variable tested. When any of the aforementioned effects had a significant impact, a standard least square model for effects’ leverage analysis was applied to identify at which value (split value) the effect could be split to create a sub-category. Hence, the variables were re-tested with MANOVA within the sub-category to confirm the absence of any significant influence of the model’s effects. Mean ± SD, median, and range for each measurement were calculated, with or without the sub-category inclusion, as necessary. Significance was set at *P* ≤ 0.05.

## Data Availability

The data analysed during the current study are available from the corresponding author on reasonable request.

## Abbreviations

CaS: Caudal site
CrS: Cranial site
DWT: Deep wall thickness
IJV: Internal jugular vein
LDVVD: Dorsoventral vein diameter on longitudinal scan
LMVD: Lateromedial vein diameters on transverse scan
MANOVA: Multivariate analysis of variance
MdS: Mid site
SD: Standard deviation
SWT: Superficial wall thickness
TDVVD: The dorsal-ventral vein diameter on transverse scan
US: Ultrasonographic
VD: Vein diameter
VP: Vein perimeter
WT: Wall thickness
